# Rapid Regression of Choroidal Metastasis under First-Line Osimertinib Therapy in Epidermal Growth Factor Receptor-Mutated Non-Small Cell Lung Cancer: A Case Report

**DOI:** 10.1159/cop/acaag008

**Published:** 2026-09-07

**Authors:** Hinrich J. Hufnagel, Oliver Zeitz, Farris Abdula, Antonia M. Joussen, Lynn S. zur Bonsen

**Affiliations:** a Department of Ophthalmology, Charité – Universitätsmedizin Berlin, Corporate Member of Freie Universität Berlin and Humboldt-Universität zu Berlin, Berlin, Germany; b Department of Ophthalmology, Medizinische Universität Lausitz – Carl Thiem, Cottbus, Germany

**Keywords:** Choroidal metastasis, Lung cancer, Epidermal growth factor receptor, Tyrosine kinase inhibitor

## Abstract

**Introduction:**

Choroidal metastases may cause substantial visual impairment in patients with metastatic non-small cell lung cancer (NSCLC). Although local ocular treatments are frequently considered, targeted systemic therapy may provide effective control in tumors harboring actionable mutations. We report the rapid regression of a choroidal metastasis following first-line osimertinib therapy in a patient with epidermal growth factor receptor (EGFR)-mutated NSCLC.

**Case Presentation:**

A 59-year-old man with metastatic EGFR-mutated NSCLC presented with progressive visual decline in the left eye. Ophthalmic examination revealed a peripapillary choroidal lesion associated with serous retinal detachment. Optical coherence tomography demonstrated subretinal fluid, consistent with a choroidal metastasis. First-line osimertinib therapy had been initiated 12 days before presentation at our clinic. An early reduction in subretinal fluid was observed during follow-up, followed by complete resolution at the 2-month follow-up. This was accompanied by regression of the choroidal lesion and improvement in visual function. No local ocular treatment was required.

**Conclusion:**

First-line osimertinib may induce rapid regression of choroidal metastases in patients with EGFR-mutated NSCLC. In selected cases, an early response to systemic targeted therapy may allow local ocular treatment to be avoided.

## Introduction

Choroidal metastases represent the most common form of intraocular malignancy and are typically associated with advanced systemic cancer, most frequently originating from breast or lung primaries [1, 2]. Their presence often signifies disseminated disease and may lead to visual impairment due to associated subretinal fluid (SRF) and retinal detachment. Management of choroidal metastases remains challenging, as no standardized treatment guidelines exist, and therapeutic strategies must be individualized based on systemic disease status, symptoms, and molecular tumor characteristics.

In recent years, advances in molecular oncology have significantly altered the therapeutic landscape of metastatic non-small cell lung cancer (NSCLC). In particular, the identification of actionable driver mutations, such as epidermal growth factor receptor (EGFR) alterations, has enabled the use of targeted therapies with improved efficacy and tolerability compared to conventional chemotherapy. Third-generation EGFR tyrosine kinase inhibitors (TKIs), such as osimertinib, demonstrate enhanced central nervous system penetration and have shown promising activity against metastatic lesions in sanctuary sites [3].

Despite increasing reports of systemic efficacy, data on the response of intraocular metastases to targeted therapies remain limited. This case highlights the rapid regression of a choroidal metastasis and restoration of visual function following initiation of osimertinib in a patient with EGFR-mutated metastatic NSCLC, underscoring the potential role of systemic targeted therapy as a noninvasive treatment option for ocular involvement. The CARE Checklist has been completed by the authors for this case report, attached as online supplementary Material (for all online suppl. material, see https://doi.org/10.1159/cop/acaag008).

## Case Presentation

A 59-year-old man was referred to our tertiary ophthalmology center on October 29, 2025, for evaluation of a nonpigmented choroidal mass in the left eye. The patient had experienced progressive visual deterioration in the left eye over the preceding 2–3 months.

At an outside institution, the lesion had been suspected to represent a choroidal metastasis in the setting of newly diagnosed NSCLC. At the time of presentation, the patient had documented pleural and splenic metastases. Molecular testing revealed an EGFR mutation, and systemic therapy with the third-generation TKI osimertinib was initiated on October 17, 2025. The patient reported subjective visual improvement shortly after treatment initiation.

### Clinical Findings

At the time of presentation in our clinic, decimal best-corrected visual acuity was 0.5 (=20/40 Snellen equivalent) in the left eye and 1.25 (=20/16 Snellen equivalent) in the right eye. Intraocular pressure was within normal limits in both eyes.

Slit-lamp examination revealed quiet anterior segments bilaterally with incipient cataracts. Fundus examination of the right eye was unremarkable.

In the left eye, funduscopy demonstrated a peripapillary nonpigmented lesion with accompanying serous-exudative retinal detachment and a mottled (“tiger-striped”) appearance in the superonasal quadrant, consistent with choroidal metastasis (shown in Fig. 1a). Optical coherence tomography revealed both central and peripheral SRF accumulation and a characteristic irregular “lumpy-bumpy” configuration of the retinal pigment epithelium (shown in Fig. 1c, d).

**Fig. 1.**
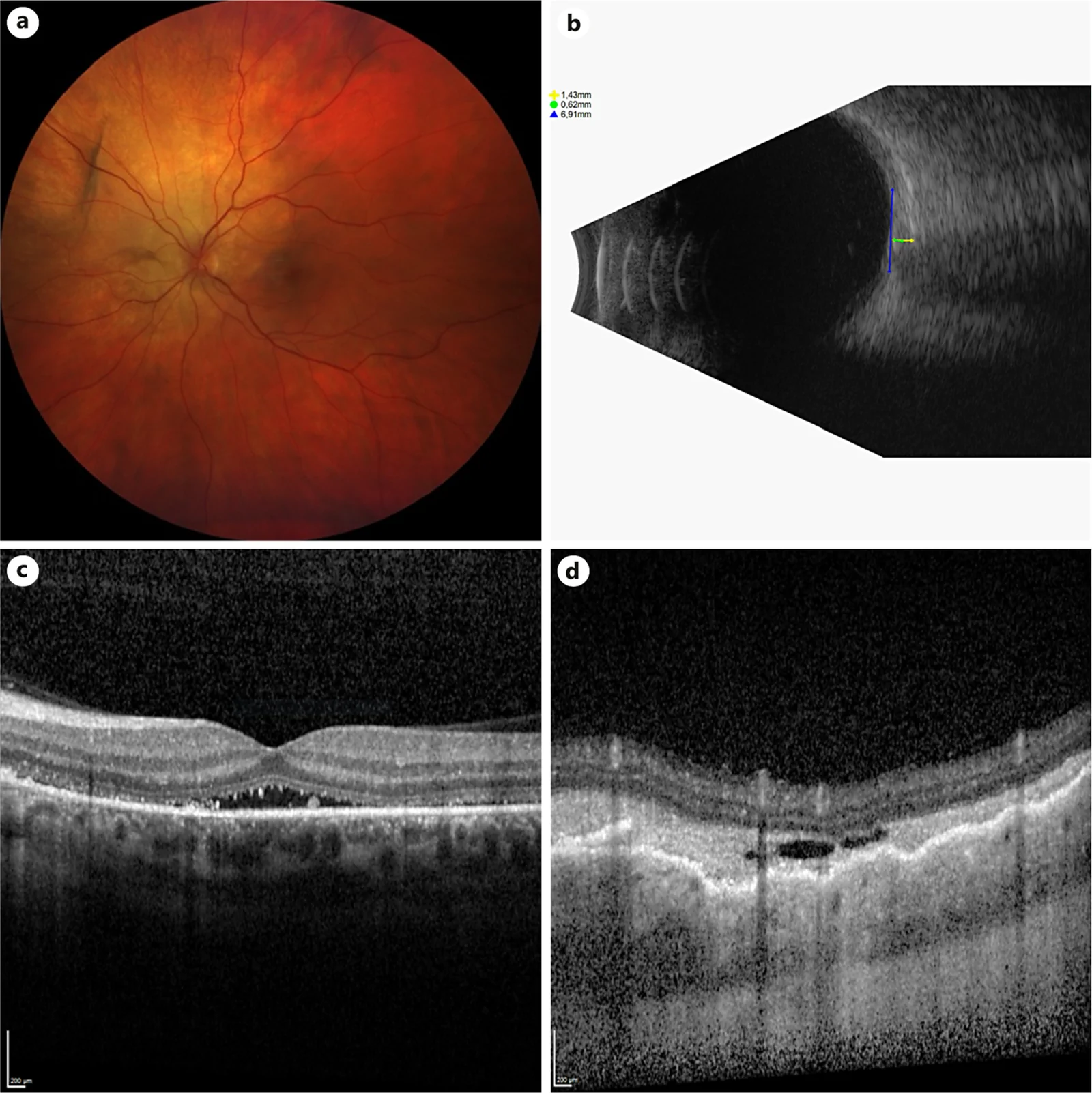
Imaging at first presentation to our outpatient department on October 29, 2025. **a** Fundus photograph of the left eye showing a peripapillary nonpigmented choroidal lesion with a mottled pattern in the superonasal quadrant. **b** B-scan ultrasonography of the left eye demonstrating a peripapillary elevation measuring 1.43 mm including the sclera and 0.62 mm excluding the sclera, with a basal diameter of 6.91 mm. **c** Macular optical coherence tomography (OCT) demonstrating central subretinal fluid (SRF). **d** Peripheral OCT scan through the lesion revealing a characteristic “lumpy-bumpy” configuration.

B-scan ultrasonography showed a moderately reflective choroidal mass with low prominence and a maximal thickness of 0.62 mm (measured from the scleral surface) (shown in Fig. 1b). Compared with imaging obtained at the outside institution on October 17, 2025 (prior to systemic therapy), there was already a marked reduction in exudation and SRF at the time of presentation (shown in Fig. 2a, b).

**Fig. 2.**
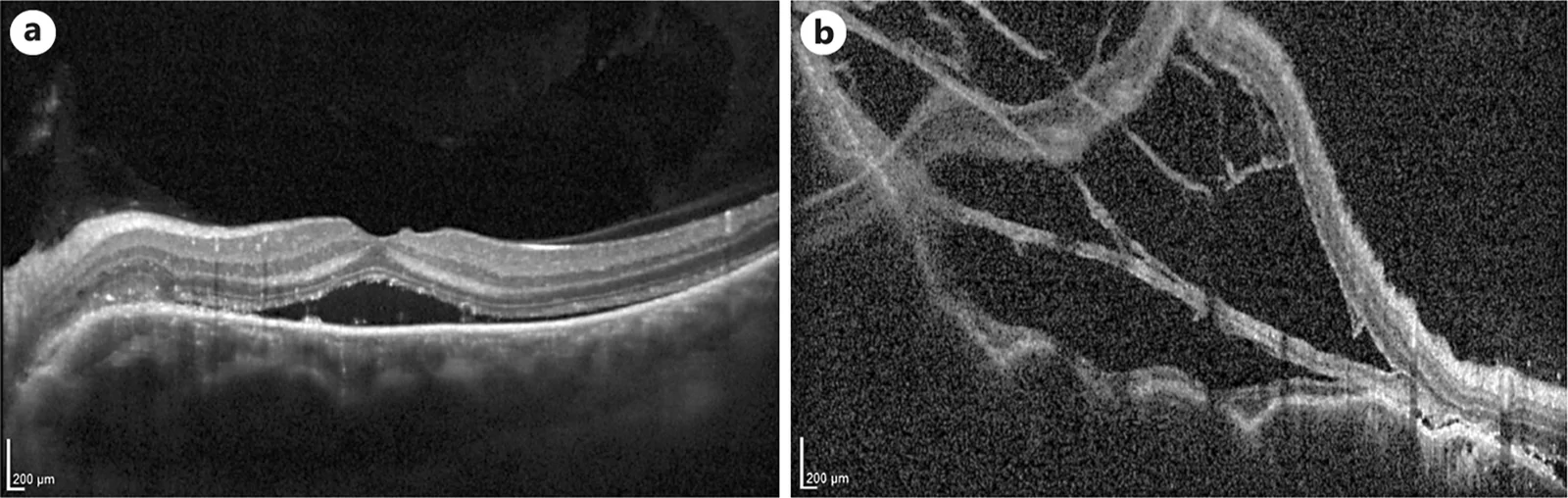
**a** Macular optical coherence tomography (OCT) of the left eye at initial ophthalmic presentation (October 17, 2025) demonstrating a prominent neurosensory detachment due to central accumulation of subretinal fluid (SRF). **b** Peripheral OCT scan in the superonasal quadrant showing retinal splitting consistent with serous fluid accumulation.

### Diagnosis

Based on the clinical and imaging findings, we diagnosed a choroidal metastasis of the left eye in the context of EGFR-mutated metastatic NSCLC.

### Treatment and Clinical Course

Systemic therapy with osimertinib had been initiated 12 days prior to presentation. Compared with baseline findings from the referring institution, we observed both subjectively perceived and objectively documented visual improvement, accompanied by significant regression of the exudative retinal detachment.

Given the rapid response, we elected to pursue a conservative approach without additional local therapy and scheduled follow-up after approximately 2 months. At 2-month follow-up, further improvement was documented. Decimal best-corrected visual acuity in the left eye improved to 1.0 (=20/20 Snellen equivalent). Optical coherence tomography imaging demonstrated complete resolution of SRF both centrally and peripherally, along with regression of the previously observed “lumpy-bumpy” configuration (shown in Fig. 3, 4). No local radiotherapy or intravitreal therapy was required.

**Fig. 3.**
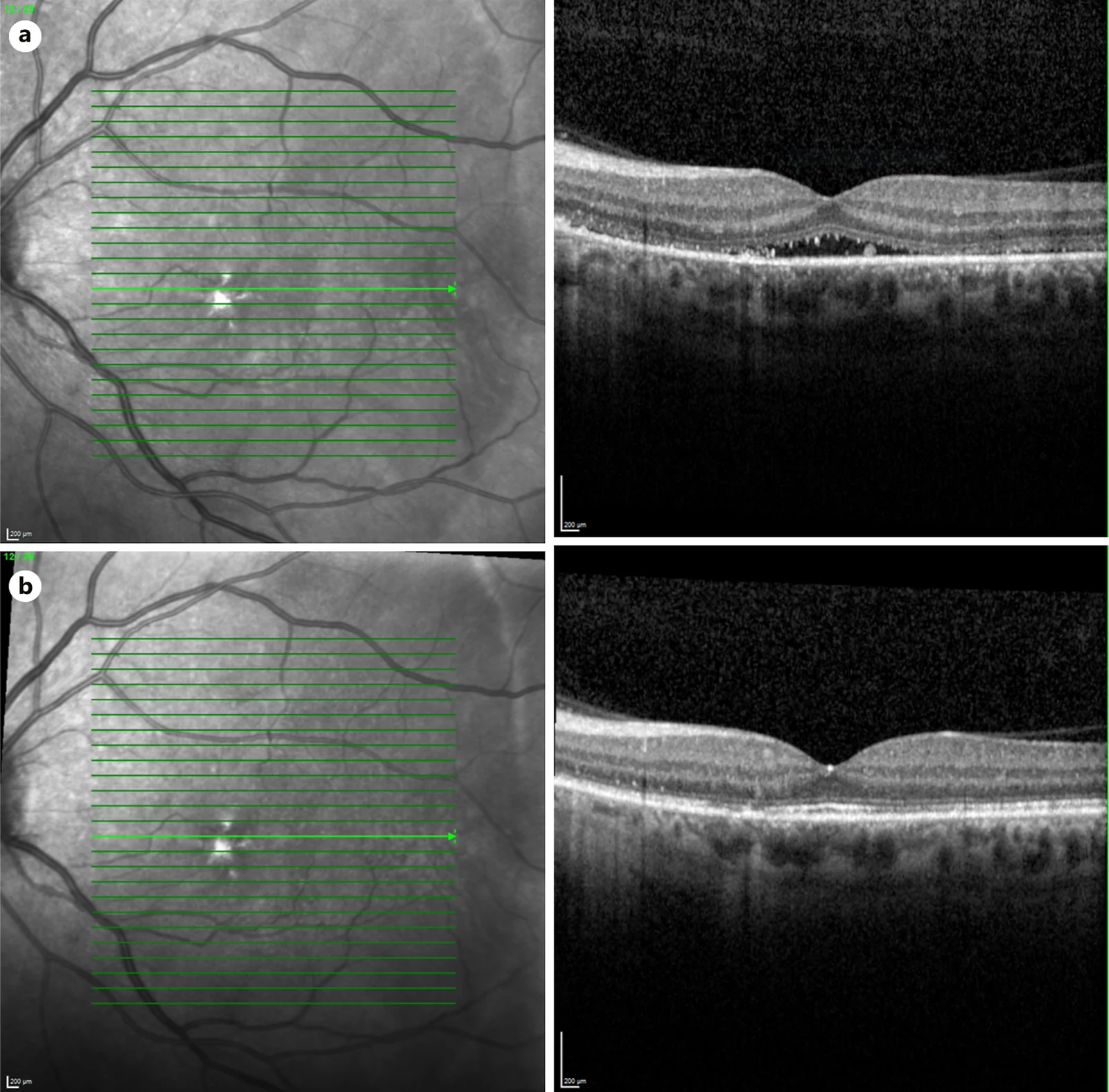
**a** Macular optical coherence tomography (OCT) obtained on October 29, 2025, demonstrating central neurosensory detachment due to accumulation of subretinal fluid (SRF). **b** Complete resolution of subretinal fluid on macular OCT obtained on December 16, 2025.

**Fig. 4.**
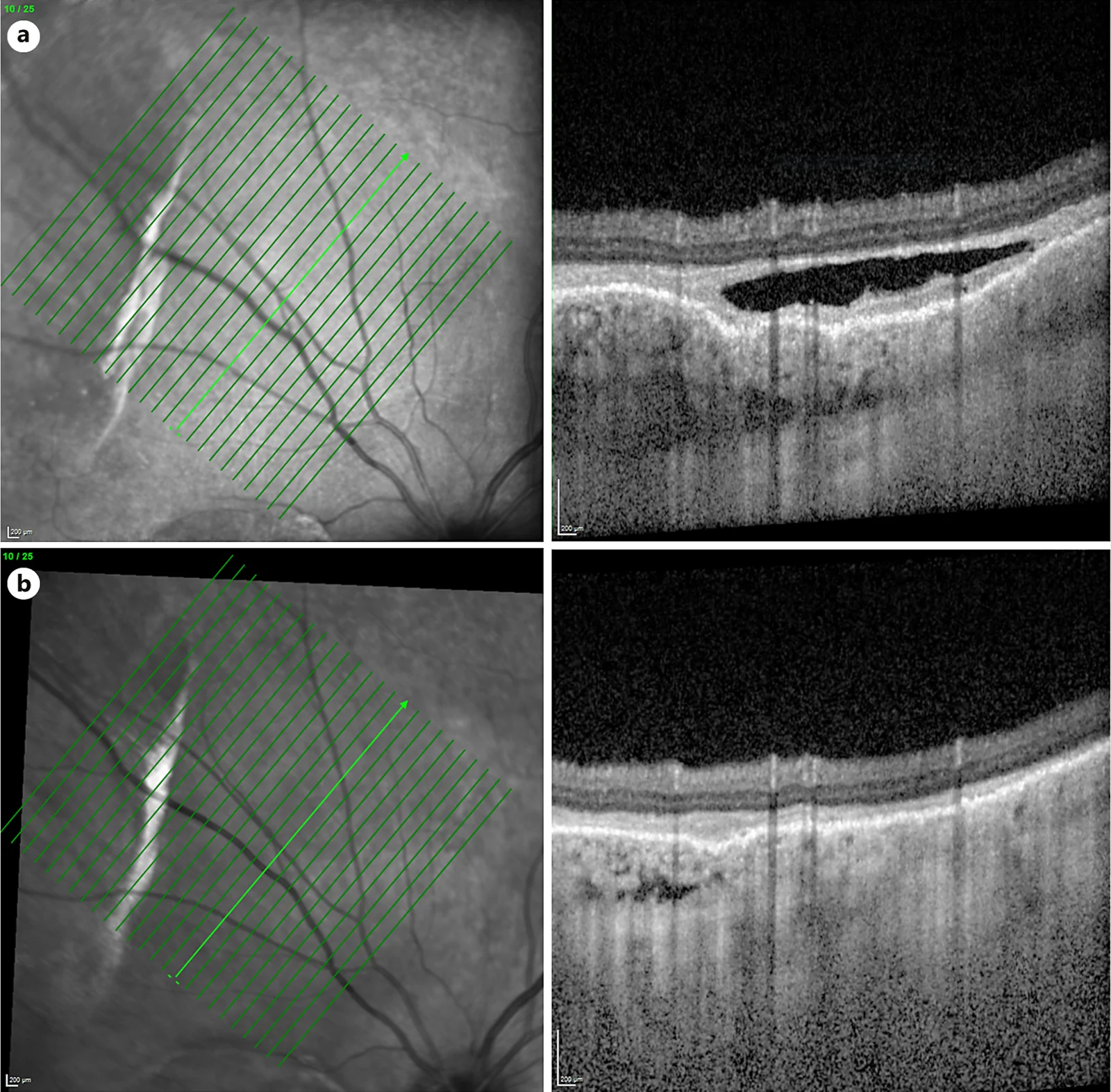
**a** Peripheral optical coherence tomography (OCT) scan obtained on October 29, 2025, through the lesion, demonstrating a “lumpy-bumpy” appearance with subretinal fluid (SRF) accumulation. **b** Regression of these findings on the corresponding OCT scan obtained on December 16, 2025.

## Discussion

Intraocular metastases are the most common intraocular malignancies. It is estimated that they are present in approximately 8–10% of patients with disseminated malignant disease [1]. A large postmortem study found intraocular metastases in as many as 12.6% of patients who died of cancer [4]. Intraocular metastases are almost exclusively found in the uveal tract, with the choroid accounting for approximately 88% of cases [2]. Because the eye is an immune-privileged organ and lacks lymphatic vessels, metastatic spread to intraocular structures occurs predominantly via the hematogenous route and preferentially involves highly vascularized tissues such as the choroid [5]. The vast majority of choroidal metastases originate from breast or lung cancer [2].

To date, no standardized therapeutic algorithm exists for choroidal metastases. Their detection indicates advanced systemic malignancy and requires an individualized, interdisciplinary approach to evaluation and treatment.

Molecularly targeted systemic therapies have significantly changed the treatment landscape. Current guidelines for advanced NSCLC recommend molecular stratification and targeted first-line therapy in the presence of actionable driver mutations, typically in stage IV disease. In approximately 10–15% of NSCLC cases in Western populations, activating EGFR mutations can be identified. Compared with platinum-based chemotherapy, EGFR-TKIs demonstrate superior efficacy and a more favorable side-effect profile [6].

The present case illustrates that systemic therapy with a third-generation TKI can be highly effective not only for systemic disease control but also for intraocular metastases. Similar to our findings, several published case reports describe rapid regression of choroidal metastases under osimertinib, often within weeks of therapy initiation [7–11]. Field et al. reported a 68-year-old patient whose initial presentation with ocular symptoms led to the diagnosis of underlying NSCLC. After initiation of osimertinib, complete resolution of SRF associated with the choroidal metastasis was observed within 1 month [7].

Other reports likewise describe partial to complete visual recovery accompanied by significant tumor regression. Importantly, durable long-term local control has also been documented. In one reported case with 5 years of follow-up, continuous systemic therapy including the second-generation EGFR-TKI afatinib followed by osimertinib as second-line treatment and pemetrexed plus adjunctive intravitreal bevacizumab as third-line therapy resulted in complete regression of the choroidal lesion, full restoration of visual function, and sustained stability throughout the observation period [11].

While these reports establish the efficacy of osimertinib, our case provides additional clinically relevant information regarding the timing of treatment response. In contrast to previously published first-line osimertinib cases, our patient demonstrated both subjective visual improvement and objectively confirmed regression of subretinal fluid only 12 days after treatment initiation. Although rapid responses have also been reported with second-line osimertinib after acquisition of the EGFR T790M resistance mutation [8, 10], evidence regarding such an early response in the first-line setting remains limited. Furthermore, the close temporal correlation between multimodal ophthalmic imaging findings and functional visual recovery highlights the value of early ophthalmologic assessment. In our patient, the documented early response directly influenced clinical management by supporting a conservative approach without additional local ocular treatment.

In a retrospective cohort of EGFR-mutant NSCLC, choroidal metastases were uncommon but frequently symptomatic and showed good local response to EGFR-TKI therapy. Notably, overall survival did not differ significantly between patients with and without ocular involvement, suggesting that choroidal metastases may not independently be associated with a worse prognosis [12].

The exceptionally early ocular response observed in our patient, with both subjective visual improvement and objectively documented anatomical regression only 12 days after treatment initiation, may be explained by the pharmacokinetic properties of osimertinib, including its lipophilicity and superior penetration of the blood-brain barrier compared with other EGFR-TKIs [3]. These characteristics may likewise explain its effectiveness in treating intraocular metastatic disease, given the physiological similarities between the blood-brain and blood-ocular barriers.

In conclusion, osimertinib represents a noninvasive therapeutic option for choroidal metastases in EGFR-mutated NSCLC and may obviate the need for local treatments such as radiotherapy. Beyond confirming its efficacy, our case demonstrates that clinically meaningful anatomical and functional improvement may already be detectable within less than 2 weeks after initiation of first-line therapy. Early multimodal ophthalmologic follow-up may therefore help guide clinical decision-making and support conservative management in appropriately selected patients.

## Statement of Ethics

This study was conducted in accordance with the tenets of the Declaration of Helsinki. Ethical approval was not required for this study, as it presents a single clinical case and does not include experimental interventions or procedures beyond routine clinical care, in accordance with institutional policies. The patient provided written informed consent for inclusion in this case report and for the publication of clinical data and images. All information has been anonymized, and no identifiable personal data are included in this manuscript.

## Conflict of Interest Statement

The authors have no conflicts of interest to declare.

## Funding Sources

This study was not supported by any sponsor or funder.

## Author Contributions

Hinrich J. Hufnagel: conceptualization, data curation, investigation, visualization, writing – original draft. Oliver Zeitz and Antonia M. Joussen: investigation, supervision, writing – review and editing. Farris Abdula: visualization, writing – review and editing. Lynn S. zur Bonsen: conceptualization, data curation, investigation, supervision, writing – review and editing.

## Data Availability

All data generated or analyzed during this study are included in this article and its online supplementary material. For further information, please contact the corresponding author.

## References

[1] Gerba-Górecka K , Romanowska-Dixon B, Karska-Basta I, Cieplińska-Kechner E, Nowak MS. Clinical characteristics and management of ocular metastases. Cancers (Basel). 2025;17(6):1041. 10.3390/cancers17061041 PMC11940828 40149375

[2] Shields CL , Shields JA, Gross NE, Schwartz GP, Lally SE. Survey of 520 eyes with uveal metastases. Ophthalmology. 1997;104(8):1265–76. 10.1016/s0161-6420(97)30148-1 9261313

[3] Colclough N , Chen K, Johnström P, Strittmatter N, Yan Y, Wrigley GL, . Preclinical comparison of the blood-brain barrier permeability of osimertinib with other EGFR TKIs. Clin Cancer Res. 2021;27(1):189–201. 10.1158/1078-0432.CCR-19-1871 33028591

[4] Eliassi-Rad B , Albert DM, Green WR. Frequency of ocular metastases in patients dying of cancer in eye bank populations. Br J Ophthalmol. 1996;80(2):125–8. 10.1136/bjo.80.2.125 PMC505402 8814741

[5] Westerwick D , Driever F, Le Guin CHD, Schmid KW, Metz KA. Intraocular metastases. Pathologe. 2017;38(6):500–6. 10.1007/s00292-017-0373-y 28993861

[6] Leitlinienprogramm Onkologie (Deutsche Krebsgesellschaft, Deutsche Krebshilfe, AWMF) . S3-Leitlinie Prävention, Diagnostik, Therapie und Nachsorge des Lungenkarzinoms. 2025 [accessed 2026 Feb 21]. Available from: https://www.leitlinienprogramm-onkologie.de/leitlinien/lungenkarzinom/

[7] Field MG , Boldt HC, Abu Hejleh T, Binkley EM. Successful response to first-line treatment with osimertinib for choroidal metastasis from EGFR-mutated non-small-cell lung cancer. Am J Ophthalmol Case Rep. 2022;26:101459. 10.1016/j.ajoc.2022.101459 PMC8899229 35265776

[8] Mariachiara M , Celeste R, Federico F, Nicole B, Antonio C. Choroidal metastasis from non-small-cell lung cancer responsive to osimertinib: a case report: efficacy of a third-generation epidermal growth factor tyrosine kinase inhibitor. Int Ophthalmol. 2018;38(6):2669–75. 10.1007/s10792-017-0749-2 29071523

[9] N Vu A , Mehta UV, Israelsen P, Ignatius Ou S-H, Browne AW. Treatment of choroidal metastasis from epidermal growth factor mutant non-small cell lung cancer with first-line osimertinib therapy. J Ophthalmic Vis Res. 2022;17(1):130–4. 10.18502/jovr.v17i1.10178 PMC8850857 35194503

[10] Keshwani K , Roelofs KA, Hay G, Lewis R, Plowman N. Treating choroidal metastases and improving vision with osimertinib in EGFR T790M-Mutated lung adenocarcinoma: a case report and review of the literature. Ocul Oncol Pathol. 2021;7(1):26–30. 10.1159/000510602 PMC7989730 33796513

[11] Nakashima K , Demura Y, Tada T, Ishizuka T. Five-year Follow-Up of choroidal metastasis from lung adenocarcinoma harboring epidermal growth factor receptor (EGFR) mutation: a case report and literature review. Cureus. 2024;16(8):e66537. 10.7759/cureus.66537 PMC11381099 39246871

[12] Bouchez C , Pluvy J, Soussi G, Nguenang M, Brosseau S, Tourne M, . Epidermal growth factor receptor-mutant non-small cell lung cancer and choroidal metastases: long-term outcome and response to epidermal growth factor receptor tyrosine kinase inhibitors. BMC Cancer. 2020;20(1):1186. 10.1186/s12885-020-07630-6 PMC7712981 33272243

